# Sensorimotor speech disorders in Parkinson's disease: Programming and execution deficits

**DOI:** 10.1590/S1980-5764-2016DN1003007

**Published:** 2016

**Authors:** Karin Zazo Ortiz, Natalia Casagrande Brabo, Thais Soares C. Minett

**Affiliations:** 1 Specialist, Master and PhD in Human Communication Disorders. Postdoctoral Fellow in Neuroscience. Associate Professor, Department of Human Communication Sciences, Universidade Federal de São Paulo, SP, Brazil.; 2 Speech Therapist, Master in Sciences, Department of Human Communication Sciences, Universidade Federal de São Paulo, SP, Brazil.; 3 Neurologist, PhD. Department of Radiology, University of Cambridge, Institute of Public Health, University of Cambridge.

**Keywords:** Parkinson's disease, motor disorders, speech disorders, dysarthria

## Abstract

**Introduction:**

Dysfunction in the basal ganglia circuits is a determining factor in the physiopathology of the classic signs of Parkinson's disease (PD) and hypokinetic dysarthria is commonly related to PD. Regarding speech disorders associated with PD, the latest four-level framework of speech complicates the traditional view of dysarthria as a motor execution disorder. Based on findings that dysfunctions in basal ganglia can cause speech disorders, and on the premise that the speech deficits seen in PD are not related to an execution motor disorder alone but also to a disorder at the motor programming level, the main objective of this study was to investigate the presence of sensorimotor disorders of programming (besides the execution disorders previously described) in PD patients.

**Methods:**

A cross-sectional study was conducted in a sample of 60 adults matched for gender, age and education: 30 adult patients diagnosed with idiopathic PD (PDG) and 30 healthy adults (CG). All types of articulation errors were reanalyzed to investigate the nature of these errors. Interjections, hesitations and repetitions of words or sentences (during discourse) were considered typical disfluencies; blocking, episodes of palilalia (words or syllables) were analyzed as atypical disfluencies. We analysed features including successive self-initiated trial, phoneme distortions, self-correction, repetition of sounds and syllables, prolonged movement transitions, additions or omissions of sounds and syllables, in order to identify programming and/or execution failures. Orofacial agility was also investigated.

**Results:**

The PDG had worse performance on all sensorimotor speech tasks. All PD patients had hypokinetic dysarthria.

**Conclusion:**

The clinical characteristics found suggest both execution and programming sensorimotor speech disorders in PD patients.

## RESUMO

**Introdução:**

Na doença de Parkinson (DP) a disfunção dos circuitos dos núcleos da base é um fator determinante na fisiopatologia dos sinais clássicos da DP e a disartria hipocinética é uma das manifestações da doença. No que se refere aos distúrbios da fala associados à DP, os modelos recentes de processamento de fala complicam a visão antiga da disartria como um déficit apenas de execução motora. Baseado nos achados de que as disfunções nos gânglios basais podem causar alterações de fala e que os distúrbios não estão apenas relacionados aos déficits de execução motora, mas também de programação motora, o objetivo deste estudo foi investigar a presença de distúrbios sensórios-motores da programação motora além dos de execução motora já descritos na fala de pacientes com DP.

**Métodos:**

O estudo é transversal e se baseou numa amostra composta por 60 adultos pareados por sexo, idade e escolaridade: 30 adultos diagnosticados com DP idiopática e 30 adultos sadios (grupo controle). Dados obtidos em um estudo prévio que analisou alterações de fluência em indivíduos com DP foram reanalisados acrescentando-se todos os tipos de manifestações/erros na fala, a fim de verificar falhas de programação e/ou execução motora. Os pacientes também realizaram avaliação da apraxia orofacial.

**Resultados:**

Todos os pacientes tinham disartria hipocinética. O grupo com DP obteve pior desempenho em todas as tarefas de fala.

**Conclusão:**

As características clínicas das manifestações/erros de fala encontrados em pacientes com DP são sugestivas de déficits de execução e de programação motora.

## INTRODUCTION

Parkinson's disease (PD) is characterized by a degeneration of neurons in the substantia nigra of the mesencephalon, leading to a fall in dopamine production. Dysfunction in the basal ganglia circuits is a determining factor in the physiopathology of the classic signs, and hypokinetic dysarthria is commonly related to PD.^1^

Regarding speech disorders associated with PD, the latest four-level framework of speech sensorimotor control^2^ proposed complicates the traditional view of dysarthria as just a motor execution disorder. This model proposes different phases of the transformation of speech code involving the different neural structures. These phases are identified as linguistic-symbolic planning, which is a nonmotor (or premotor) process, motor planning, motor programming and execution. According to the cited author,^2^ Linguistic Symbolic Planning is the phase where linguistic rules of language are involved and this level of processing is nonmotor in nature so typical symptoms are aphasia signs. During the Motor planning phase a gradual transformation of symbolic units (phonemes) into a code that can be handled by the motor system takes place. Speech signs and symptoms resulting from disorders in motor planning can include slow, struggling speech with distortions and even apparent substitutions. Motor programming is a phase that determines the spatiotemporal and force dimensions such as the amount of muscle tension needed, velocity, direction and range. A disorder at this level can result in impairment in these aspects and repeated initiation. Finally, during the execution phase, the hierarchy of plans and programs is finally transformed into non-learned automatic motor adjustments.^2^

 The role of the structures such as the basal ganglia and the lateral cerebellum in both motor programming and execution suggests the possibility of dual symptomatology in certain types of dysarthria, particularly in the parkinsonian (hypokinetic) type.

It is well known that the circuits in the basal ganglia play a fundamental role in the mechanisms of stuttering commonly present in these patients.^3^ It is important to recognize that neurogenic stuttering is totally different from the other kinds of stuttering. Disfluencies in PD patients may be analogous to limb motor symptoms such as difficulty with the initiation of motor movements and festination of gait observed in walking.^4^ In this case, these failures could be more related to programming than execution deficits. In a previous study,^5^ a Speech Fluency Assessment Protocol^6^ was applied to classify typology of disruptions into typical or atypical disfluencies. The atypical disfluencies such as repetitions of syllables; repetition of sounds; prolongation; blocking; pauses (over two seconds) and intrusions of sounds or segments and episodes of palilalia, characterized by the presence of repetitions of syllables (over four times) and words (over three times), with or without acceleration of speech rate were analysed. The authors found that PD subjects had a significantly higher number of speech disfluencies overall compared to control subjects. In light of this, most of the characteristics described by the authors might be related to motor programming problems, especially considering the current view that the most prominent disfluency type in PD is sound repetition, followed by initial syllable and word repetitions and some prolongations.^7^ In order words, some of these characteristics could be analysed as programming deficits. Apraxia of speech is believed to result from a motor planning deficit. In a previous study on apraxia of speech in PD, the authors found that half of the PD patients presenting dysarthria also had apraxia of speech.^8^

Another approach is the use of Nonspeech Assessment for understanding the speech production mechanism. Darley et al.,^9^ in their presentation of Motor Speech Disorders, recommended several nonspeech observations and maneuvers during the assessment. There is continuing debate over the utility of nonspeech tasks for informing clinical diagnosis.^10^ According to Ballard et al.,^11^ studies have reached different conclusions. The authors stated that, nonspeech tasks can provide useful information about the functioning of the motor system. A study investigating the association between speech and orofacial apraxia found an association in 48% of cases studied.^12^ Although the classification of these speech disorders differed to that currently in use, the possibility of an association between these two conditions cannot be ruled out.

Based on findings that dysfunctions in basal ganglia can cause fluency of speech deficits, and on the premise that the speech deficits seen in PD are not related to an execution motor disorder alone but also to a disorder at the motor programming level, the main objective of this study was to investigate the presence of sensorimotor disorders of programming (besides the execution disorders previously described) in PD patients. 

## METHODS

This study was approved by the Research Ethics Committee of the *Universidade Federal de São Paulo* (protocol number 0843\09). All participants signed a free and informed consent form.

Casuistic. A cross-sectional study was conducted in a sample of 60 adults matched for gender, age and education: 30 adult patients diagnosed with idiopathic PD attended at the Sector for Motor Disorders of the Neurology Department of the *Universidade Federal de São Paulo*, and 30 healthy adults (control group) that were companions or family members of the patients assessed. 

The general inclusion criteria for both groups were as follows: age ≥ 50 years; education ≥ 4 years; absence of personal or family history of developmental or psychogenic stuttering or language disorders; absence of history of stroke or previous traumatic brain injury; absence of alcoholism or use of illegal drugs; visual or hearing impairments which could affect performance on the tasks given; normal performance on the MMSE for educational level, according to the standards established for the Brazilian population,^13^ thus excluding subjects with dementia from the sample and ensuring that impairments in cognitive aspects did not interfere with the specific assessment.

The patients participating in the study were diagnosed with PD, had not undergone neurosurgery, were at stages 2, 2.5 or 3 on the Hoehn & Yahr,^14^ and in use of medication for PD. Thus, subjects at initial or advanced stages of the disease were excluded from the sample because individuals at the initial stage may not have impaired speech while, in advanced cases, speech samples may be unintelligible or insufficient. 

All patients were at the 'on' phase of the medication during the assessment.

Instruments. First, the patients were submitted to the Protocol for Dysarthria Assessment.^15^ Respiration, phonation, articulation, resonance and prosody were evaluated in order to check for the presence of Hypokinetic Dysarthria.

For the sensorimotor speech disorders assessment, the subjects told a story based on sequences of pictures composed of seven drawings and also described a typical day to produce a sufficient speech sample for subsequent analysis. 

The oral agility subtest of the Boston Diagnostic Aphasia Examination (BDAE) was used to evaluate speech and orofacial praxis.^16^ This test includes six tasks of orofacial agility and seven involving speech agility. The orofacial agility task comprises oral commands such as tongue to alternate corners of the mouth, protrude and retract tongue, tongue alternately to upper and lower teeth, purse lips and release, open and close mouth, retract and release lips. The subject must perform the movements correctly in terms of programming and timing. On the speech agility task, the subject has to repeat words as fast as they can in a correct fashion. The score is given according to correct repetition and timing. Speech errors were analysed using the same criteria as presented below.

Data collection was carried out on an individual basis. The discourse produced was recorded using a digital camera (SONY Cyber - shot 6.0 mega pixels) and later transcribed. The data were obtained from a sample of a previous study^5^ in which fluency disorders were analysed. In that study, episodes of palilalia, number of hesitations; interjections; revisions; unfinished words; repetition of words, segments and sentences, repetitions of syllables; repetition of sounds; prolongation; blocking; pauses and intrusions of sounds or segments and also speech rate, were analyzed as fluency disorders.

In the present study, all types of articulation errors were reanalyzed to investigate the nature of these errors. In this new analysis, interjections, hesitations, repetitions of words or sentences (during discourse) were considered typical disfluencies; blocking, episodes of palilalia (words or syllables) were analysed as atypical disfluencies. We analysed features including successive self-initiated trial, phoneme distortions, self-correction, repetition of sounds and syllables, prolonged movement transitions, addition or omissions of sounds and syllables, all of which can be related to programming disorders of sensorimotor control of speech. It is noteworthy that successive self-initiated trial, phoneme distortions, addition and omission can also been found in planning disorders. The features present on each test were scored with 1 point. Total score was calculated by summing all feature scores.

Statistical analysis. Categorical data were compared using the Chi-squared (c^2^) test (without Yates comparison) with application of Fisher's exact test when Cochran's restrictions were present.

A probability (p) of less than 0.05 was considered statistically significant and all tests were two-tailed. Differences among means were calculated for a ninety-five percent confidence interval (95%CI). All statistical analyses were carried out using the software SPSS (Statistical Package for the Social Science) version 11.5.1 for Windows.

## RESULTS

Forty patients with PD, attended at the Sector for Motor Disorders of the Department of Neurology of the *Universidade Federal de São Paulo,* were scheduled for speech assessment. Of this total, 10 were not included in the sample because they did not attend the scheduled session. Thus, a total of 30 patients followed the protocol, in addition to 30 controls. The data from these 60 subjects were considered in the subsequent analyses.

General characteristics. The age of subjects in the sample ranged from 50 to 75 years, with a mean age 62.3±7.0 years, and in terms of gender, 82% were men. 

There were no statistically significant differences between the Control group (CG) and the Parkinson's disease group (PDG) for age (62.4±6.9 *versus* 62.2±7.1 years; t(58)=0.13; 95%CI= -3.4 to 3.9; p=0.898), education (8.7±4.2 *versus* 8.4±4.2 years; t(58)=0.21; 95%CI= -2.0 to 2.4; p=0.832), MMSE score (28.5±1.2 *versus* 28.4±1.4; t(58)=0.29; 95%CI= -0.6 to 0.8; p=0.770) or gender (83% men *versus* 80% men; c^2^(1)=0.11; p=0.739). 

Clinical characteristics of PD patients. Disease duration ranged from 2 to 20 years (mean=9.9, SD=4.4), 20% of patients had a score of 2 on the Hoehn and Yahr scale, 37% scored 2.5 and the remainder scored 3. A total of 90% of the patients were in use of Levodopa, 37% Amantadine, 10% Selegiline, 60% Pramipexole and 13% Biperiden. Of the 30 patients in the sample, 24 (80%) were in use of combined medications whereas 6 (20%) used a single medication. Of the single users, five used levodopa and one pramipexole. 

**Figure 1:**
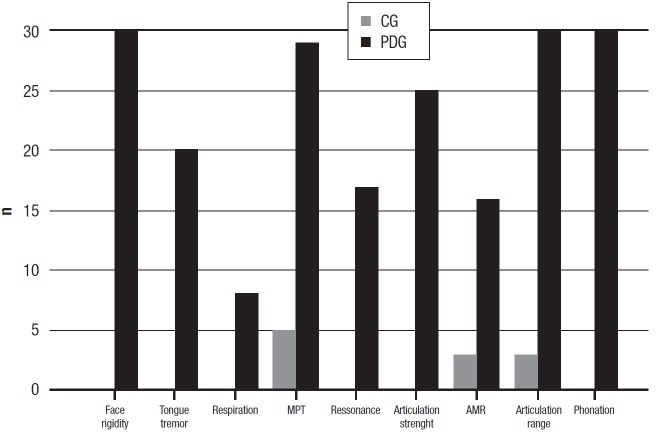
Distribution of types of changes in dysarthrias according to study group.

Dysarthria assessment results. The distribution of changes, according to study group: face rigidity, tremor of tongue, increased respiration, decreased maximum phonation time (MPT), altered resonance, reduced articulation strength, slow alternate motion rate (AMR), reduced articulation amplitude and change in voice quality are shown in Figure 1.

The CG had significantly better performance than the PDG for all dysarthria features (1.1±0.7 versus 6.7±1.3; t(58)= -20.2; 95%CI= -6.0 to -4.9; p<0.001).

Assessment of non-verbal and verbal praxis. The CG had significantly better performance than the PDG for both non-verbal (7.6±1.8 versus 4.9±1.6; t(58)=5.88; 95%CI=1.76 to 3.57; p<0.001) and verbal (12.0±0.6 versus 11.0±1.1; t(58)=4.26; 95%CI=0.53 to 1.47; p<0.001) praxis. 

For the purposes of intragroup comparison of two types of apraxia, we calculated the proportion of correct responses on the oral agility tests of each individual to standardize the results. 

This comparison revealed that the proportion of correct responses on the task assessing verbal praxis was significantly higher than on the tasks assessing non-verbal praxis in both groups.

- CG: 0.63±0.15 versus 0.86±0.04; t(29)= -8.47; 95%CI= -0.28 to -0.17; p<0.001
- PDG: 0.41±0.14 versus 0.37±0.14 years; t(29)= -15.09; 95%CI= -0.42 to -0.32; p<0.001

The total features found for spontaneous speech in the CG was significantly lower than in the PDG (4.8±2.6 versus 8.9±6.7; t(58)= -3.12; 95%CI= -6.7 to 1.4; p=0.003). The CG had significantly better performance than the PDG on the verbal agility task from the Boston test (12.0±0.6 versus 11.0±1.1; t(58)=4.26; 95%CI=0.53 to 1.47; p<0.001).

## DISCUSSION

The most relevant finding of this study was that analysis of all features of speech clearly suggested impairments at the motor programming and execution level in the patients with Parkinson's disease. 

The idea of reanalyzing separately all types of errors had the principal goal of identifying the occurrence of programming disorders.

In relation to dysarthria, only AMR, MPT and reduced range of articulation were seen in some CG individuals (Figure 1). The finding of these alterations in a few individuals may be related to aging. In the PD group, alterations were observed in all motor bases and it was clearly possible to statistically differentiate the two groups. All PD patients presented dysarthria.

In Table 1, it can be observed that different speech errors were more evident in the PD group. Speech errors were identified in three speech samples: telling the story, describing a day, and the agility task of the Boston test.

**Table 1:** Statistical data for groups studied according to speech characteristics.

SPEECH FEATURES IN CG AND PDG									
	CG					PDG			
	Mean	SD	Min	Max		Mean	SD	Min	Max
Typical Disfluencies	0.84	1.11	0	5		0.76	1.16	0	5
Atypical disfluencies	0	0	0	0		1	2.11	0	8
Self-correction	0.53	0.78	0	3		0.43	0.77	0	3
Self-initiated trials	0.17	0.46	0	2		0.1	0.31	0	1
Prolonged movement transitions	0	0	0	0		0.93	1.66	0	8
Repetition of syllables	0.03	0.18	0	1		0.2	0.48	0	2
Repetition of sounds	0	0	0	0		0.07	0.37	0	2
Phoneme distortions	0	0	0	0		0.23	0.57	0	2
Addition of sounds	0	0	0	0		0.1	0.31	0	1
Omissions of sounds	0	0	0	0		0	0	0	0

SD: standard deviation; min: minimum; max: maximum.

An analysis of errors committed on the oral agility task showed that eight of the 30 patients from the PDG had motor programming of speech deficits, not observed in the control group. During this task, syllable repetition was the only feature present in the PDG. Although the syllable repetition featured by the PDG patients can be present in both neurogenic stuttering and speech apraxia (nowadays regarded as a motor planning disorder), making it hard to differentiate between the conditions, some considerations should be taken into account. First, stuttering associated with acquired neurological disorders can mask the presence of other communication problems.^7^ Over the years, various subgroups of neurogenic stuttering have been proposed, such as differentiations between dysarthric stuttering, apraxic stuttering and dysnomic stuttering.^17^ More recently, further subdivisions have been suggested based on underlying lesion location^18^ and stuttering associated with extrapyramidal disease has been described.^19^ On this point, the most prominent disfluency type is sound repetition and in this study we found more syllable repetition, features more related to programming disorders. However, accurately distinguishing between these syndromes remains challenging.

Some authors^20^ in a study review affirmed that, although the onset of stuttering in fluent speaking adults has been discussed in the literature for over a century, it remains unclear whether acquired stuttering is a distinct disorder or an epiphenomenon of speech deficits such as apraxia of speech. Although the exact nature of repetition is unclear, in this case it would be considered, according to the latest four-level framework of speech sensorimotor control, a programming disorder and not a planning disorder. 

Besides, given that patients performed the test more slowly, other speech errors may not have been manifested on this task.

Previous studies that considered apraxia a programming disorder, state that the speech deficits occurring in PD are not related only to the muscle control level, causing dysarthria, but also to the speech programming level, with the condition of apraxia.^8^ In the cited study, the apraxic patient comprised a subgroup of a group with dysarthria, leading authors to believe that perhaps speech apraxia does not exist in PD without being associated with dysarthria. The authors concluded that dysarthria is twice as frequent as apraxia in PD. In our study, we found that all patients presented dysarthria and some presented speech errors that suggested programming deficits.

Non-verbal and verbal apraxias have been previously described in other neurodegenerative diseases that occur with parkinsonian syndrome. Cases of individuals with corticobasal degeneration (CBD) and progressive supranuclear palsy (PSP) that presented impairments such as speech apraxia, non-fluent aphasia or a combination of both disorders, have been reported.^21^ The authors stated these disorders are often not detected at disease onset but become evident at more advanced stages and can be associated with pathologic diagnoses of CBD and PSP. According to the authors, patients with these neurodegenerative disorders also exhibit initial changes of speech apraxia and non-fluent aphasia and in general, the condition progresses rapidly compared to the classic picture characteristic of PD. In a study involving 35 patients with CBD, three had speech apraxia.^22^

The quest for a better understanding of the process of programming has primarily sought a comprehensive formulation of the role of the different neural structures involved in the programming phase of motor processing. The motor areas involved in motor programming comprise the basal ganglia, lateral cerebellum, supplementary motor area, motor cortex, and the frontolimbic system.^2^ It is generally accepted that the basal ganglia,^2^ ^,^ ^23^ and the lateral cerebellum^2^ ^,^ ^24^ in particular, are involved in programming, and these parts perform complementary functions.^2^ ^,^ ^25^ The exact role of each, however, is not yet fully understood.^2^ Parkinson's disease causes delayed initiation, slowed execution, abnormal sequential complex movements and an inability to automatically execute learned motor plans.^2^ ^,^ ^26^ Dysarthria due to Parkinson's disease also indicates that the basal ganglia may play a role in initiation, temporal synchronization, timing and automatized production of speech,^2^ ^,^ ^27^ as observed in all motor tasks analyzed in the current study. 

During the repetition tasks, we observed that eight of the 30 patients from the PDG presented symptoms such as: syllable repetitions, besides episodes of accelerated speech while performing the task, whereas controls did not. Phoneme substitutions, distorted substitutions, omissions and additions were not found in this sample, probably because motor planning was preserved in these patients.

Comparison of performance of the two groups analyzed revealed a statistically significant difference in total score obtained on the tasks for both non-verbal and verbal praxis, i.e. the PD patients had significantly worse performance on both tasks.

Intragroup comparison of the two types of apraxia revealed that the proportion of correct responses on the task assessing verbal praxis was significantly higher than on the tasks assessing non-verbal praxis in both groups. This result suggests that these tasks may be more sensitive for the early detection of cases that progress to programming disorders.

We noted that all individuals performed the movements with impaired velocity, although 15 subjects, besides slowness in performing the movements, also exhibited praxic deficits, i.e. in motor programming, evidenced by non-performance or partial performance of the movements. 

The need to demonstrate the movements, known to facilitate motor programming, was frequent in the PDG whereas CG subjects did not require this aid. Therefore, we concluded that the poorer performance seen in the PD group on the task assessing non-verbal praxis can be explained by deficits in programming and sequencing movements, i.e. non-verbal praxis. Other explanations include the difficulty in motor execution present in PD and the presence of both these deficits, as observed in 15 subjects from the PDG. Thus, it is notable that the task proposed, although originally intended to assess apraxia, was also sensitive for assessing dysarthria-related motor aspects. This was the case because velocity is one of the elements of the assessment procedure and allowed co-occurrence of apraxia and dysarthria-related motor aspects that hamper the performance of movements to be identified. 

Based on assessment of the five motor bases of speech, all patients in the sample had previously been diagnosed with hypokinetic dysarthria. Therefore, non-verbal apraxia was an impairment which occurred concomitantly with the dysarthric condition in some cases. Thus, all of the apraxic patients in this study were dysarthric but not necessarily the other way around. 

To conclude, the PDG had worse performance on all sensorimotor speech tasks. All PD patients had hypokinetic dysarthria. The clinical characteristics found suggest both execution and programming sensorimotor speech disorders in PD patients.
